# A comparison of the four healthy days measures (HRQOL-4) with a single measure of self-rated general health in a population-based health survey in New York City

**DOI:** 10.1186/s12955-020-01560-4

**Published:** 2020-09-24

**Authors:** Sarah E. Dumas, Tenzin Yangchen Dongchung, Michael L. Sanderson, Katherine Bartley, Amber Levanon Seligson

**Affiliations:** Bureau of Epidemiology Services, Division of Epidemiology, New York City Department of Health and Mental Hygiene, Queens, NY 11101 USA

**Keywords:** Health-related quality of life, Self-rated general health, Healthy days measures, Population-based health surveys, Predictive power

## Abstract

**Background:**

Data on health-related quality of life (HRQOL) can be used to track health disparities, assess the impact of chronic diseases, and predict mortality. The Centers for Disease Control and Prevention’s “Healthy Days Measures” (HRQOL-4) assesses four key domains: self-rated general health, physical health, mental health, and activity limitations. The domains are not easily combined to summarize overall HRQOL, and some evidence suggests that self-rated general health may be an adequate proxy indicator for overall HRQOL. This study compares self-rated general health as a solitary measure of HRQOL with two summary indices of the HRQOL-4 as a predictor of adverse health conditions in a representative sample of adult New York City residents.

**Methods:**

The 2017 NYC Social Determinants of Health survey implemented by the New York City Department of Health and Mental Hygiene collected data from a representative sample of New Yorkers (*n* = 2335) via phone, mail, and web. We compared the information criteria and predictive power of self-rated general health with two alternative summary indices of the HRQOL-4 in predicting self-reported health conditions (hypertension, diabetes, obesity, non-specific psychological distress, and a summary indicator for at least one those four morbidities).

**Results:**

Overall, 19.1% (95% CI: 16.9, 21.5) of respondents reported that they had fair or poor general health. Self-rated general health was significantly associated with days of poor physical health, poor mental health, and activity limitations (*p* < 0.001 for each). While the Akaike and Bayesian information criteria suggested that the summary indices of the HRQOL-4 produced marginally better models for predicting adverse health conditions, self-rated general health had slightly higher predictive power than did the summary indices in all models of physical health outcomes as measured by Tjur’s pseudo-R^2^ and the area under the curve.

**Conclusion:**

We found very small differences between self-rated general health and the summary indices of the HRQOL-4 in predicting health conditions, suggesting self-rated general health is an appropriate proxy measure of overall HRQOL. Because it can be measured with a single question rather than four, it might be the most simple, efficient, and cost-effective method of summarizing HRQOL in large population-based surveys.

## Background

Health-related quality of life (HRQOL) is a multidimensional concept that summarizes the impact of health and disease on quality of life [1, 2]. HRQOL is considered an indicator of overall health, synthesizing the key domains related to physical, mental, emotional, and social functioning [1–3]. In public health practice HRQOL data supplement traditional measures of morbidity and mortality and have been used to identify and track health needs and disparities [4–10], assess the population-level impact of chronic diseases [11–21], and predict mortality [22–24].

The Centers for Disease Control and Prevention (CDC) measures HRQOL using the core “Healthy Days Measures” (HRQOL-4). This module assesses an individual’s self-rated general health, physical health, mental health, and activity limitations through four questions:

- Q1. Would you say that in general your health is excellent, very good, good, fair, or poor?
- Q2. Now thinking about your physical health, which includes physical illness and injury, how many days during the past 30 days was your physical health not good?
- Q3. Now thinking about your mental health, which includes stress, depression, and problems with emotions, how many days during the past 30 days was your mental health not good?
- Q4. During the past 30 days, approximately how many days did poor physical or mental health keep you from doing your usual activities, such as self-care, work, or recreation?

**Fig. 1:**
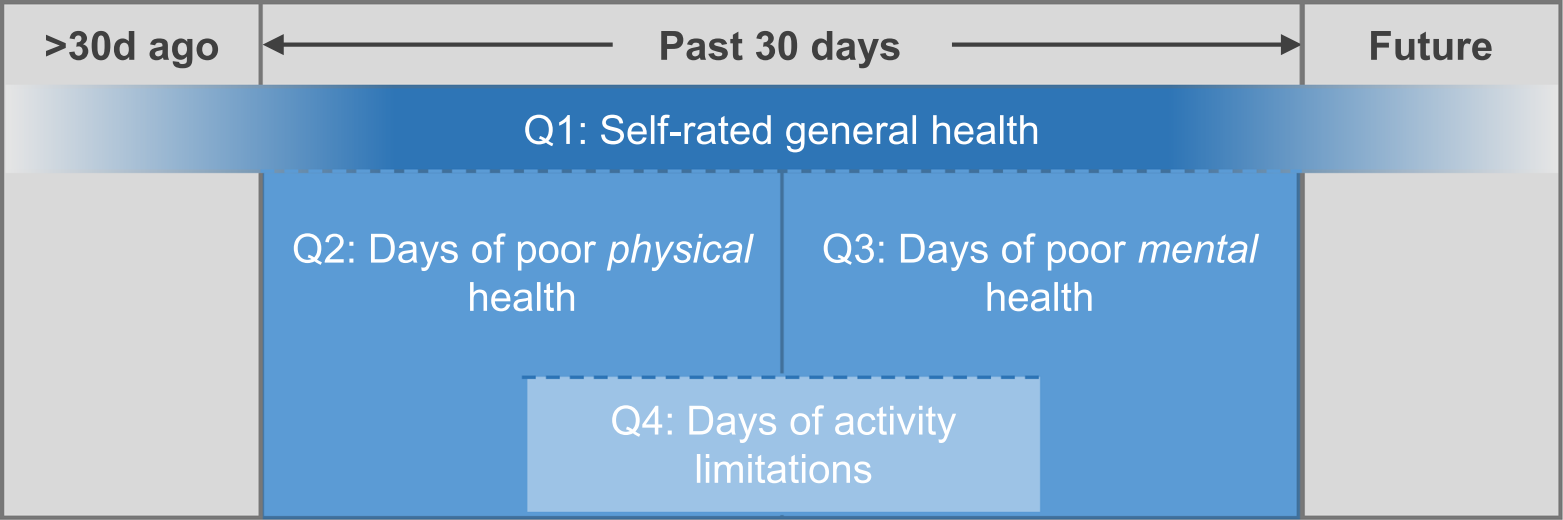
Conceptual relationship of the four items comprising the CDC Healthy Days Measures core module (HRQOL-4). Q1 captures respondents’ overall assessment of their health in the recent past, incorporating both their physical and psychological health, with some consideration for the more distant past and future. Q2 and Q3 measure physical and mental health in the past 30 days, respectively, conceptualized as independent domains. Q4 captures perceived disability or poor functional status as a result of the poor mental or physical health days measured in Q2 and Q3. Adapted with permission from Hennessy CH, et al. Public Health Rep. 1994;109(5):665–72

Each item measures a different domain, which are conceptually related (Fig. 1 [1];). Research has demonstrated that the four domains within the core module are valid and reliable in a variety of populations [1, 25–29].

Although the four HRQOL-4 domains are valuable for summarizing health, if a study finds an improvement in one item and a decline in another, it can be difficult to draw an overall conclusion about impact. Therefore, to provide a more cohesive picture of the impact of overall health on quality of life, researchers and public health practitioners have summarized the HRQOL-4 in a single variable using several different methods. First, a summary index of “unhealthy days” can be calculated by summing the number of physically unhealthy days and the number of mentally unhealthy days [3]. The unhealthy days (UHD) index is widely used by researchers [9, 10, 19–21, 27, 30, 31], but it assumes that an individual’s physical and mental health are independent constructs [3] and has not been fully validated as a summary of overall health [32].

Second, some researchers have proposed viewing HRQOL as a single latent construct that can be revealed through factor analysis [28, 29, 33, 34]. Although such models have shown goodness of fit, validity, and stability over time [33], they can be complex to develop and are not readily interpretable. Third, some researchers have attempted to quantify the presumed latent HRQOL construct by dichotomizing each of the four items and summing them to generate a score (range: 0–4) [35, 36]. While benefiting from simplicity and ease of interpretability, these models do not appear to have been validated.

Finally, the self-rated general health item can itself be considered as a proxy indicator for overall HRQOL [1], as it broadly reflects an individual’s health as they define it. Poor self-perceived general health has been found to be associated with objective measures of poor physical health and disability [23, 37–39], poor mental health [40–42], and harmful health risk behaviors [40, 43–48]. It has also been found to be a strong predictor of mortality [23, 24, 49–54]. Because self-rated health can be measured with a single question, it may be the most simple, efficient, and cost-effective method of summarizing HRQOL in large population-based surveys, and there may not be added value of asking the full HRQOL-4.

This study compares self-rated general health as a solitary measure of HRQOL with two summary indices of the HRQOL-4 as a predictor of adverse health conditions in a representative sample of adult New York City (NYC) residents. Our results have broad implications for measuring HRQOL in large, population-based surveys.

## Methods

### Data source

In 2017, the NYC Department of Health and Mental Hygiene launched the Social Determinants of Health survey, a health survey of adults (≥ 18 years of age) in NYC. It was a multimode, multiframe survey, incorporating random-digit dialing and address-based sampling. The telephone sample was composed of a random selection of NYC cell (40,575) and landline (47,625) telephone numbers. The address-based sample came from the United States Postal Service Computerized Delivery Sequence File, which contained a complete listing of all NYC residential addresses, from which a geographically representative sample of 6152 units was selected. Respondents from the telephone frame responded via phone; respondents from the address-based frame were invited to respond either by mail or web. Telephone interviews were conducted in English, Spanish, Russian, Mandarin, and Cantonese, while mail and web interviews were available only in English.

The survey (*n* = 2335; 1433 responded by phone, 247 by web, and 655 by mail) had an overall response rate of 11.6% and cooperation rate of 80.4%, using modified versions of the American Association for Public Opinion Research’s (AAPOR) Response and Cooperation Rates #3 [55]. For the phone frame (Response Rate #3 = 6.2%), response and cooperation rates were calculated for a dual-frame (cell and landline) random-digit dial survey. For the address-based sample (Response Rate #3 = 20.3%), response and cooperation rates were calculated using a modified version of AAPOR’s response rate calculation for mail surveys to unnamed persons. The combined address-based and phone response rate was calculated by weighting each frame’s share of the total number of completed interviews.

Data were weighted to the adult residential population of New York City per the American Community Survey 2015, adjusting for the probability of selection, frame overlap, and nonresponse. Estimates were age-adjusted to the US 2000 standard population. The survey methodology has been described in detail elsewhere [56].

All procedures and data collection instruments for the Social Determinants of Health survey were reviewed and approved by the Department of Health and Mental Hygiene Institutional Review Board. The protocol for the current analysis was additionally reviewed by the Institutional Review Board and determined to be exempt from further oversight.

### Measures

The single-item self-rated general health question from the HRQOL-4 (Q1) is a five-level ordinal variable, scored such that 1 indicates “excellent” health and 5 indicates “poor” health. The other three items from the HRQOL-4 (Q2, Q3, and Q4) were analyzed as continuous variables, as has been done previously [32].

Two summary indices of HRQOL-4 were generated. First, the UHD index was calculated by summing the number of physically and mentally unhealthy days, capped at 30 total days [3]. For some analyses, the UHD was then dichotomized such that 0 = less than 14 days and 1 = greater than or equal to 14 days of poor overall health. Second, we dichotomized each of the four items in the HRQOL-4 and summed them to generate a simple summary score (SSS), expressed as a binary variable. For this calculation, general health status was dichotomized such that 0 = excellent, very good or good health, and 1 = fair or poor health. Physically unhealthy days, mentally unhealthy days, and days of activity limitation were dichotomized such that 0 = less than 14 days of unhealthy days and 1 = greater than or equal to 14 days of poor health or activity limitations. The four dichotomized variables were then summed (range 0–4) and further dichotomized as “good health” if scored from 0 to 2 and “poor health” if scored 3–4 to generate the final SSS binary variable (Supplementary Materials 1).

The four self-reported measures of morbidity were: 1) hypertension; 2) diabetes; 3) obesity (a binary variable generated from body mass index [BMI], calculated using self-reported height and weight, where obesity is BMI ≥30); and, 4) serious nonspecific psychological distress (a binary variable calculated from responses to six items in the Kessler-6, a validated screening instrument where serious nonspecific psychological distress is Kessler-6 ≥ 13 [57]). Finally, a binary variable was generated indicating if the individual reported having at least one of these four health conditions.

Covariates included in models were based on self-reports and included measures of age, sex, race/ethnicity, and educational attainment.

### Analytical approach

Weighted prevalence estimates and 95% confidence intervals were used to describe the sample characteristics.

To investigate construct validity, we fit five linear and logistic models of self-rated general health, adjusted for age group, race/ethnicity, sex, and educational attainment, with the three other domains of the HRQOL-4 and the two summary indices of the HRQOL-4 as the primary predictors of interest. We then estimated the weighted conditional margins (or estimated marginal means) to provide predictions of the five response variables at each of the five levels of self-rated health after controlling for the covariates. In the case of the binary outcome, the SSS index, the conditional margins estimated the predicted probability of being categorized as having “poor health” in the past 30 days by the SSS index at each level of self-reported general health. Overall F-tests measures the significance (*p* < 0.05) of the association between self-rated general health and the response variables.

To measure the association between the measures of overall HRQOL (self-rated general health, UHD index, and SSS index) and morbidity (hypertension, obesity, diabetes, nonspecific psychological distress, and any morbidity), we fit multivariable logistic regression models and generated adjusted odds ratios (OR). For this analysis, self-rated health was dichotomized, where fair or poor health was modelled against good, very good, or excellent self-rated health. The UHD index was also dichotomized, where 0 = less than 14 days and 1 = greater than or equal to 14 days of poor overall health. Models were adjusted for age group, race/ethnicity, sex, and educational attainment. To assess differences in model quality and power in predicting individual health outcomes between the three summary measures of overall HRQOL, we calculated the Akaike information criterion (AIC), the Bayesian information criterion (BIC), Tjur’s pseudo-R^2^, and the area under the curve (AUC).

The AIC and BIC were used to compare the relative quality of the three models for each outcome based on their goodness of fit and parsimony. Tjur’s pseudo-R^2^ and AUC were calculated to compare the predictive power of the three HRQOL measures for each morbidity outcome. Tjur’s pseudo-R^2^, also called the coefficient of discrimination [58], is calculated as the absolute value of the difference in the mean predicted probabilities for the success and failure of an event [59]. The statistic varies between 0 and 1, with 1 indicating perfect predictive power. The AUC, also known as the concordance index or c-statistic, is a measure of discrimination interpreted as the estimated probability that, under the fitted model, a randomly selected person with the outcome (e.g., hypertension) will have a higher predicted probability of having the outcome than a person who does not have the outcome [60]. The AUC varies from 0.5, indicating no discrimination, to the theoretical maximum of 1. A model with an AUC between 0.5 and 0.7 is considered to have poor discrimination, 0.7 to 0.8 to have acceptable discrimination, and above 0.8 to have excellent discrimination [60].

All analyses were conducted using SAS Enterprise Guide version 7.15 and SUDAAN version 11.0.1.

## Results

### Participant characteristics and measures of HRQOL and morbidity

Participant characteristics and measures of HRQOL and morbidities are presented in Table 1. Overall, one-fifth of respondents reported having fair 14.2% (95% CI: 12.3, 16.4%) or poor 4.8% (3.7, 6.2%) health. On average, respondents reported 4.5 (4.0, 5.0) days of poor physical health, 4.6 (4.1, 5.1) days of poor mental health, and 3.4 (3.0, 3.9) days of activity limitations in the past 30 days. Participants reported an average of 7.9 (7.2, 8.5) unhealthy days in the past 30 days as measured by the UHD index. The SSS index calculated that 8.0% (6.5, 9.9%) of respondents were in poor health. The prevalence of morbidities ranged from 30.0% (27.5, 32.6%) of NYC adults with hypertension to 6.5% (5.3, 8.0%) who had nonspecific psychological distress. Nearly half of respondents reported having at least one of the four conditions measured in the survey.

**Table 1:** Participant demographic characteristics, health-related quality of life measures, and self-reported morbidities

Demographics	n	Weighted % (95% CI)
Age, years (mean, 95% CI)	2259	45.3 (44.3, 46.3)
Female (%, 95 CI)	2331	54.0 (51.1, 56.9)
Race/ethnicity (%)	2335	
White, non-Latino		34.4 (31.8, 37.2)
Black, non-Latino		22.2 (19.9, 24.7)
Latino		27.5 (25.0, 30.2)
Asian/Pacific Islander		13.4 (11.5, 15.6)
Other, non-Latino		2.4 (1.7, 3.4)
Educational attainment (%, 95 CI)	2315	
Less than high school degree		18.8 (16.3, 21.6)
High school degree		24.2 (21.7, 26.8)
Some college		23.2 (20.7, 25.7)
College degree or more		33.9 (31.5, 36.4)
**Heath-related quality of life items**		
General health status (%, 95 CI)	2324	
Excellent		19.0 (16.9, 21.3)
Very good		29.3 (26.7, 32.0)
Good		32.6 (30.0, 35.4)
Fair		14.2 (12.3, 16.4)
Poor		4.8 (3.7, 6.2)
Days of poor physical health, past 30 days (mean, 95% CI)	2272	4.5 (4.0, 5.0)
Days of poor mental health, past 30 days (mean, 95% CI)	2278	4.6 (4.1, 5.1)
Days of activity limitations, past 30 days (mean, 95% CI)	2285	3.4 (3.0, 3.9)
**Overall health-related quality of life summary indices**		
UHD index (mean, 95% CI)	2253	7.9 (7.2, 8.5)
SSS index (%, 95 CI “poor health”)	2219	8.0 (6.5, 9.9)
**Morbidities**		
Self-reported morbidities (%, 95 CI)		
Hypertension	2330	30.0 (27.5, 32.6)
Obesity	2243	25.6 (23.1, 28.3)
Diabetes	2323	11.7 (10.0, 13.7)
Nonspecific psychological distress, past 30 days	2330	6.5 (5.3, 8.0)
Summary of self-reported morbidities (%, 95 CI)	2228	
None		52.0 (49.0, 54.9)
At least one morbidity		48.0 (45.1, 51.0)

*Abbreviations*: *UHD* Unhealthy days, *SSS* Simple summary score

### Is self-rated health associated with the other domains of the HRQOL-4 and two summary indices of the HRQOL-4?

In adjusted models, a graded relationship was observed between self-rated health and the other measures of HRQOL-4, with higher days of poor physical health and mental health, days of activity limitations, days of poor overall health (UHD index), and probability of poor health (SSS index) associated with incrementally worse self-rated general health (Table 2 and Supplementary Materials 2). On average, after controlling for covariates, participants who reported excellent, very good, or good health had few days of poor physical health, poor mental health, and activity limitations. They also had a relatively low UHD index and had a low predicted probability of being in poor health by the SSS index. In contrast, participants reporting fair general health spent over half of the past 30 days with poor physical and/or mental health (UHD) on average, while those reporting poor general health spent nearly 90% of the past 30 days with poor physical or mental health on average. Both groups also had a high probability of being categorized as having “poor health” by the SSS index. Overall, after controlling for covariates, days of poor physical health, days of poor mental health, and days of activity limitations, overall days of poor heath (UHD index), and probability of having poor health as measured by the SSS index increased incrementally as self-rated general health worsened. Self-rated general health was significantly associated with all five indicators of HRQOL (*p* < 0.001 for each; Table 2).

**Table 2:** Weighted conditional margins estimating the mean number of days of poor physical health, days poor mental health, days of activity limitations, overall unhealthy days (UHD index), and probability of poor health as measured by a simple summary score (SSS index) at each level of self-rated general health, 2017 NYC Social Determinants of Health Survey

	Self-rated general health					***p*** value
	Excellent	Very Good	Good	Fair	Poor	
Days of poor physical health, past 30 days (mean days, 95% CI; *n* = 2213)	1.2 (0.7, 1.8)	1.9 (1.4, 2.3)	3.8 (3.1, 4.5)	10.0 (8.2, 11.8)	22.1 (19.8, 24.5)	< 0.001
Days of poor mental health, past 30 days (mean days, 95% CI; *n* = 2219)	1.7 (1.0, 2.5)	2.9 (2.2, 3.6)	5.0 (4.2, 5.9)	7.3 (5.9, 8.8)	16.4 (13.4, 19.3)	< 0.001
Days of activity limitations, past 30 days (mean days, 95% CI; *n* = 2225)	0.8 (0.4, 1.3)	1.0 (0.6, 1.3)	3.2 (2.5, 3.8)	7.8 (6.1, 8.7)	17.3 (14.3, 20.3)	< 0.001
UHD index (mean days, 95% CI; *n* = 2195)	2.7 (1.9, 3.6)	4.4 (3.7, 5.2)	8.0 (6.9, 9.1)	15.5 (13.5, 17.5)	26.4 (24.7, 28.2)	< 0.001
SSS index (%, 95 CI; n = 2219)^a^	0.2 (0.0, 1.2)	0.2 (0.0, 1.2)	2.0 (1.0, 4.1)	28.7 (21.7, 36.9)	72.7 (61.0, 82.0)	< 0.001

*Abbreviations*: *UHD* Unhealthy days, *SSS* Simple summary score

^a^ Predicted probability of someone categorized as having “poor health” by the SSS index falling into each of the five self-rated health categories

### Which of the three alternative measures of HRQOL provide the best model fit and highest predictive power of five morbidity outcomes?

Having “poor health” as measured by self-rated general health, the UHD index, or the SSS index was associated with significantly greater odds of reporting hypertension, obesity, diabetes, nonspecific psychological distress, and at least one of these conditions (Table 3). These associations are particularly strong for nonspecific psychological distress.

**Table 3:** A comparison of self-rated general health with two summary indices of health-related quality of life (HRQOL) on five health outcomes, using adjusted odds ratios (OR), information criteria, and measures of predictive power, 2017 NYC Social Determinants of Health Survey

	Outcome				
	Hypertension	Obesity	Diabetes	Nonspecific psychological distress, past 30 days	At least one morbidity
**Self-rated general health**	OR: 2.30 (1.64, 3.23)	OR: 2.97 (2.11, 4.18)	OR: 3.14 (2.08, 4.74)	OR: 8.74 (5.55, 13.76)	OR: 4.23 (2.85, 6.27)
Fair/poor self-rated general health	AIC: 6038500	AIC: 6300947	AIC: 3682550	AIC: 2560623	AIC: 7051256
	BIC: 6038678	BIC: 6301124	BIC: 3682728	BIC: 2560801	BIC: 7051433
	pseudo-R^2:^ 0.2535	pseudo-R^2:^ 0.0925	pseudo-R^2:^ 0.1557	pseudo-R^2:^ 0.0947	pseudo-R^2:^ 0.2051
	AUC: 0.801	AUC: 0.695	AUC: 0.807	AUC: 0.754	AUC: 0.765
	*n* = 2262	*n* = 2185	*n* = 2257	*n* = 2261	*n* = 2173
**UHD index**	OR: 1.45 (1.03, 2.04)	OR: 1.65 (1.18, 2.32)	OR: 2.24 (1.46, 3.44)	OR: 17.35 (9.83, 30.30)	OR: 2.37 (1.67, 3.36)
≥ days of poor physical health	AIC: 5980053	AIC: 6272465	AIC: 3555416	AIC: 2221851	AIC: 7031422
	BIC: 5980230	BIC: 6272642	BIC: 3555593	BIC: 2222028	BIC:7031599
	pseudo-R^2:^ 0.2362	pseudo-R^2:^ 0.0719	pseudo-R^2:^ 0.1353	pseudo-R^2:^ 0.1288	pseudo-R^2:^ 0.1888
	AUC: 0.793	AUC: 0.682	AUC: 0.787	AUC: 0.844	AUC: 0.755
	*n* = 2201	*n* = 2125	n = 2195	*n* = 2200	*n* = 2115
**SSS index**	OR: 2.20 (1.32, 3. 66)	OR: 2.22 (1.33, 3.71)	OR: 1.76 (1.04, 2.98)	OR: 14.78 (8.40, 26.02)	OR: 4.88 (2.24, 9.76)
Poor HRQOL	AIC: 5826778	AIC: 6140378	AIC: 3516550	AIC: 2328617	AIC: 6935581
	BIC: 5826955	BIC: 6140555	BIC: 3516727	BIC: 2328795	BIC: 6935757
	pseudo-R^2:^ 0.2427	pseudo-R^2:^ 0.0698	pseudo-R^2:^ 0.1264	pseudo-R^2:^ 0.1466	pseudo-R^2:^ 0.1865
	AUC: 0.797	AUC: 0.680	AUC: 0.783	AUC: 0.755	AUC: 0.756
	*n* = 2168	*n* = 2093	*n* = 2162	*n* = 2167	*n* = 2083

*Abbreviations*: *AIC* Akaike information criterion, *AUC* Area under curve, *BIC* Bayesian information criterion

Relative to self-rated general health and the UHD index, the SSS index produced marginally better quality models for predicting hypertension, obesity, diabetes, and at least one morbidity, but not nonspecific psychological distress, according to both the AIC and BIC (Table 3). For predicting nonspecific psychological distress, the UHD index produced the highest quality model. In contrast, self-rated general health had slightly higher predictive power relative to the two summary indices in models predicting hypertension, obesity, diabetes, and at least one morbidity as measured by Tjur’s pseudo-R^2^ and AUC. However, in models of nonspecific psychological distress, the SSS index (as measured by Tjur’s pseudo-R^2^) and the UHD index (as measured by the AUC) had greater predictive power. All models demonstrated good predictive properties for predicting hypertension, diabetes, nonspecific psychological distress, and at least one morbidity, but had poor discrimination for the obesity outcome.

## Discussion

A single survey question measuring self-rated general health status was significantly associated with the three other domains comprising the HRQOL-4: the number of days that respondents experienced poor physical health, poor mental health, and activity limitations. It was also associated with two summary indices combining all four domains of the HRQOL-4, the UHD and SSS indices. Self-rated general health was associated with five physical and mental health indicators, just as were the two UHD and SSS indices; however, neither the UHD nor the SSS predicted health outcomes better than a single question about general health status. AIC and BIC measures of fit were only slightly better for the UHD and SSS indices than self-rated general health in models of the five health outcomes, and Tjur’s pseudo-R^2^ and AUC measures indicated that general health status was a better predictor of physical health outcomes than the summary indices. Only for predicting non-specific psychological distress in the past 30 days did the Tjur’s pseudo-R^2^ and AUC measures indicate better fits for the SSS and UHD index than the single question on general health status.

While the summary indices had marginally better model fit than the single measure of general health status for all of the health outcomes, the single measure had higher predictive power than the summary indices for all health outcomes except non-specific psychological distress. This has important implications for cost and efficiency. Survey costs depend on mode, sample size, and the complexity of the study design [61]. Another cost associated with surveys is respondent time and burden. Designing surveillance instruments must balance collecting needed data with parsimony by including only questions needed to answer research questions [62, 63]. Surveys can substantially reduce respondent burden and survey costs by asking one question rather than four.

Notably, self-rated general health had poor model fit and predictive power relative to the summary indices in models of nonspecific psychological distress. This finding is consistent with previous research, which has shown that self-rated general health is not as strongly associated with mental health compared with physical health. For example, Horner-Johnson et al. (2009) found in a factor analysis that the general health item loaded only on the physical health factor and was only weakly correlated to the mental health factor [28]. Similarly, Ounpuu et al. (2000) found that 50.3% of respondents reporting greater than 7 days of poor mental health in the past 30 days also indicated that their general health was very good or excellent [64]. This suggests that respondents’ assessment of general health may be more strongly influenced by their perceptions of their physical health and highlights an important potential limitation for the use of self-rated general health as a summary of overall HRQOL. Given the concern that self-rated health may capture physical health better than mental health [28, 64–66], this analysis should be replicated with additional mental health indicators.

This analysis had several limitations. First, only four health outcomes were used to evaluate the relative predictive power of self-rated general health and the UDH and SSS indices. Hypertension [67], obesity [68], diabetes [69], and nonspecific psychological distress are key indicators of adult population health, but these four indicators alone may not sufficiently capture overall health. Potential indicators that could be tested in future research include: a history of cancer or heart disease; elevated blood cholesterol; kidney disease; asthma, chronic obstructive pulmonary disease (COPD), and other respiratory diseases; unhealthy behaviors such as binge drinking, smoking [69], and sedentary lifestyle; dementia; and depression [70].

A second limitation was that the analysis was conducted on a sample of NYC adults overall, rather than on particular populations of adults. Some research has suggested that observed differences in self-rated general health across racial and ethnic groups in the United States may be due to different conceptions of the idea of “health” based in culture and language [71–73]. Future analyses should replicate this in larger samples to explore the validity of self-rated health as a measure of HRQOL across racial and ethnic groups.

This study did not aim to assess the validity of the components of the HRQOL in and of themselves. Future work could implement an approach such as the COSMIN Study Design Checklist [74] to assess content validity, structural validity, internal consistency, and other elements.

The data were based on a sample of NYC adults, and survey response bias may have limited the extent to which the data are representative of adult New Yorkers. Participants differed from the general NYC adult population in various ways, especially when analyzed by survey mode. For example, 53.4% of respondents who took the survey via the web were White, compared to 34.4% of NYC adults overall according to the 2015 American Community Survey. In another metric, 72.9% of respondents who took the survey via the web had a college education, compared to 33.9% of New Yorkers overall. This suggests that there was sampling bias, which we attempted to mitigate using the weighting approach described above.

Limitations notwithstanding, this study had several strengths. First, the data were gathered using multiple sampling frames and modes. This should allay concerns that the single self-rated general health question may not reliably measure health under different implementation contexts. Second, the data were collected from a representative sample of a large urban population, using survey weights, and therefore findings may be generalizable to other similar populations. Additional research should be replicated in other contexts within the United States to see if the findings hold within rural and suburban populations and within specific subpopulations that program evaluations may be assessing and to detect differences by sampling frames or modes.

## Conclusion

Surveillance and program evaluation data collection efforts are costly, and many nuances of public health and programmatic efforts need to be captured along with indicators of outcomes. Public health practitioners should consider asking only one question about general health rather than four in order to summarize HRQOL so that space can be dedicated to other valuable data that need to be collected.

## Supplementary information

**Additional file 1: Supplementary Materials 1.** Illustration demonstrating how the unhealthy days (UHD; Panel A) and simple summary score (SSS; Panel B) indices of the HRQOL-4 were constructed. **Supplementary Materials 2.** Weighted estimated marginal means of days of poor physical health, days poor mental health, days of activity limitations, and unhealthy days (UHD) increases incrementally with worsening self-reported general health. Weighted predicted probability of “poor health-related quality of life” by the simple summary index (SSS) increases with worsening self-reported general health, 2017 NYC Social Determinants of Health Survey.

## Data Availability

The data supporting the conclusions of this article are available from the New York City Department of Health and Mental Hygiene with a Data Use Agreement. Please contact EpiDataRequest@health.nyc.gov for inquiries.

## Abbreviations

AIC: Akaike information criterion
AUC: Area under the curve
BIC: Bayesian information criterion
BMI: Body mass index
CDC: Centers for Disease Control and Prevention
HRQOL: Health-related quality of life
HRQOL-4: CDC’s core Healthy Days Measures
NYC: New York City
OR: Odds ratio
SSS: Simple summary score
UHD: Unhealthy days
