# Supplementary material for: A comparison of the four healthy days measures (HRQOL-4) with a single measure of self-rated general health in a population-based health survey in New York City
Source: Health Qual Life Outcomes. 2020 Sep 24;18:315. doi: 10.1186/s12955-020-01560-4 (PMC7517637; doi:10.1186/s12955-020-01560-4)
Supplement: Supplementary file 1 — Additional file 1: Supplementary Materials 1. Illustration demonstrating how the unhealthy days (UHD; Panel A) and simple summary score (SSS; Panel B) indices of the HRQOL-4 were constructed. Supplementary Materials 2. Weighted estimated marginal means of days of poor physical health, days poor mental health, days of activity limitations, and unhealthy days (UHD) increases incrementally with worsening self-reported general health. Weighted predicted probability of “poor health-related quality of life” by the simple summary index (SSS) increases with worsening self-reported general health, 2017 NYC Social Determinants of Health Survey. [file 12955_2020_1560_MOESM1_ESM.docx]

**Supplementary Materials**

**
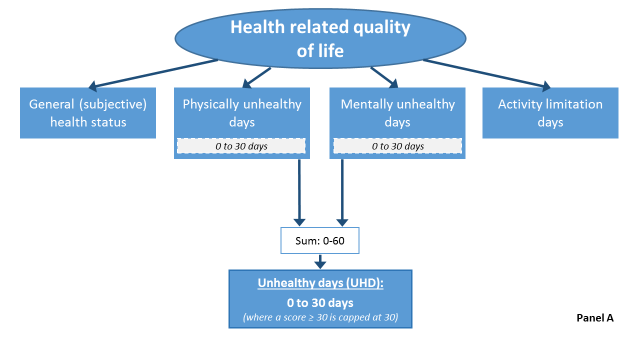
**

**
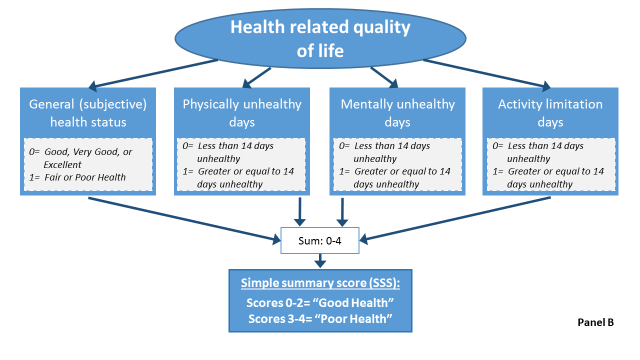
Supplementary Materials 1. Illustration demonstrating how the unhealthy days (UHD; Panel A) and simple summary score (SSS; Panel B) indices of the HRQOL-4 were constructed.**


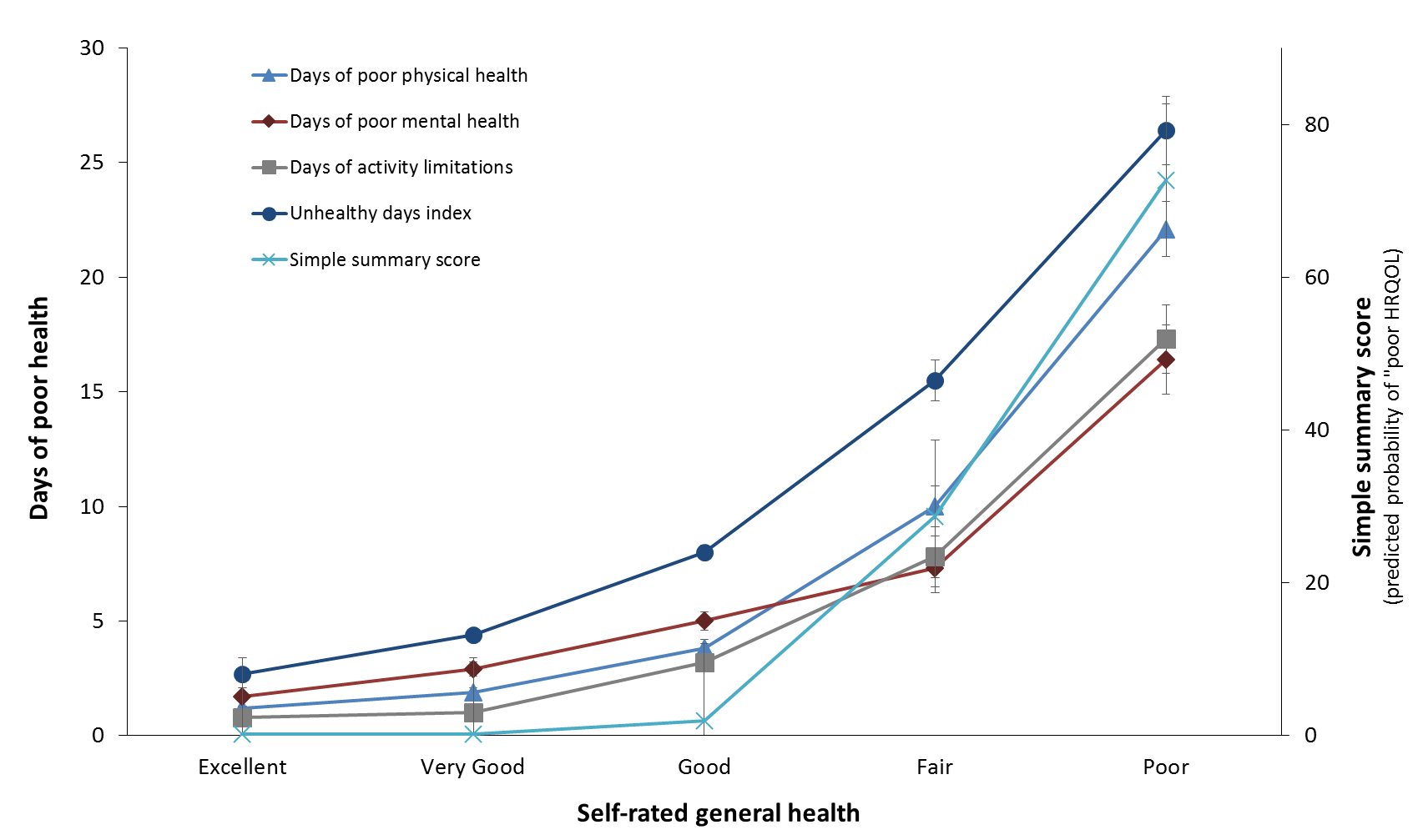


**Supplementary Materials 2. Weighted estimated marginal means of days of poor physical health, days poor mental health, days of activity limitations, and unhealthy days (UHD) increases incrementally with worsening self-reported general health. Weighted predicted probability of “poor health-related quality of life” by the simple summary index (SSS) increases with worsening self-reported general health, 2017 NYC Social Determinants of Health Survey.**
